# A pressure-reversible cellular mechanism of general anesthetics capable of altering a possible mechanism for consciousness

**DOI:** 10.1186/s40064-015-1283-1

**Published:** 2015-09-07

**Authors:** Kunjumon I. Vadakkan

**Affiliations:** Division of Neurology, Department of Medicine, University of Toronto, Sunnybrook Health Sciences Centre, 2075 Bayview Avenue, Room A4-08, Toronto, ON M4N 3M5 Canada

**Keywords:** Consciousness, General anesthetics, Pressure reversal, Semblance hypothesis, Inter-membrane contact, Membrane hemifusion, Partial hemifusion, Complete hemifusion, Membrane fusion, Neurodegeneration

## Abstract

Different anesthetics are known to modulate different types of membrane-bound receptors. Their common mechanism of action is expected to alter the mechanism for consciousness. Consciousness is hypothesized as the integral of all the units of internal sensations induced by reactivation of inter-postsynaptic membrane functional LINKs during mechanisms that lead to oscillating potentials. The thermodynamics of the spontaneous lateral curvature of lipid membranes induced by lipophilic anesthetics can lead to the formation of non-specific inter-postsynaptic membrane functional LINKs by different mechanisms. These include direct membrane contact by excluding the inter-membrane hydrophilic region and readily reversible partial membrane hemifusion. The constant reorganization of the lipid membranes at the lateral edges of the postsynaptic terminals (dendritic spines) resulting from AMPA receptor-subunit vesicle exocytosis and endocytosis can favor the effect of anesthetic molecules on lipid membranes at this location. Induction of a large number of non-specific LINKs can alter the conformation of the integral of the units of internal sensations that maintain consciousness. Anesthetic requirement is reduced in the presence of dopamine that causes enlargement of dendritic spines. Externally applied pressure can transduce from the middle ear through the perilymph, cerebrospinal fluid, and the recently discovered glymphatic pathway to the extracellular matrix space, and finally to the paravenular space. The pressure gradient reduce solubility and displace anesthetic molecules from the membranes into the paravenular space, explaining the pressure reversal of anesthesia. Changes in membrane composition and the conversion of membrane hemifusion to fusion due to defects in the checkpoint mechanisms can lead to cytoplasmic content mixing between neurons and cause neurodegenerative changes. The common mechanism of anesthetics presented here can operate along with the known specific actions of different anesthetics.

## Background

It is not yet known how different general anesthetics that function as gamma-aminobutyric acid_A_ (GABA_A_) receptor agonists, alpha adrenergic receptor agonists, *N*-methyl-d-aspartic acid (NMDA) receptor antagonists, dopamine receptor antagonists and opioid receptor agonists operate to achieve the common function of reversible loss of consciousness (Kennedy and Norman 2005; Brown et al. 2011; Kopp et al. 2009). Even though the concept of degeneracy whereby consciousness is produced by many mechanisms was introduced (Århem et al. 2003), it is not known how and where they block the neurobiological mechanism for consciousness. In this context, it is thought that the primary reason for not understanding the converging mechanism that leads to loss of consciousness is the lack of knowledge about the physiological process of consciousness (Århem et al. 2003; Beecher 1947). Once a framework for consciousness becomes available from neurobiological mechanisms, it should be able to explain a common mechanism of anesthetics. The lipophilic property of the anesthetics provides a common platform since various receptors upon which different anesthetics act are located within the lipid membranes. It is also known that the hydrophobic anesthetic molecules incorporated within the membrane can change the conformation of the receptor molecules and alter their function (Rosenberg et al. 1975; Miller and Pang 1976; Haydon et al. 1977; Trudell 1977; Vanderkooi et al. 1977; Franks and Lieb 1978). However, it is not known how the observed alteration in the functions of different types of receptors is associated with the common function of blocking consciousness.

Alternate mechanisms for anesthetic action were also put forward (Halsey et al. 1978; Mashour 2004; Hamaroff 2006). The observation that general anesthesia induced by different anesthetics can be reversed by the application of high amounts of either hydrostatic or gas phase pressure applied on the animals (Johnson and Flagler 1950; Johnson and Miller 1970; Lever et al. 1971; Miller 1974; Halsey and Wardley-Smith 1975; Kent et al. 1977; Beaver et al. 1977; Smith et al. 1984; Wann and Macdonald 1988; Daniels 2000; Chau et al. 2009) provides a challenge and an opportunity to determine the unique common mechanism of anesthetics. A theoretical framework for consciousness derived from a neurobiological mechanism that can also be extended to explain other higher brain functions and related findings at different levels can be examined for a possible mechanism of anesthetics. Different findings at different levels—such as the lipophilic nature of anesthetics, the converging function of several anesthetics disturbing the mechanism of consciousness, the reversal of the unconscious state back to the normal conscious state after withdrawal of anesthetics and the pressure reversal of anesthesia—provide a problem-set whose solution is likely to provide a unique mechanistic explanation. In this regard, the previously explained framework of consciousness (Vadakkan 2010) from the semblance hypothesis (Vadakkan 2013) is examined.

## General anesthetics

The most commonly used measurement to estimate the potency of an anesthetic in humans is the minimum alveolar concentration (MAC) that prevents gross muscular response to a surgical incision in 50 % of patients. At around 0.3–0.5 MAC, the ability to respond to verbal commands is lost in 50 % of patients, and the onset of unconsciousness is reached. Above 1.0 MAC, immobility to noxious stimulus is achieved (Hentschke et al. 2005). The currently used general anesthetics are lipophilic in nature and are more readily able to cross the blood–brain barrier. Anesthetic potency correlates with the solubility of the anesthetic chemicals in lipids and has been thought to be related to their hydrophobicity (Miller et al. 1972a, b). Since different anesthetics are known to act on specific receptors on the lipid membrane (Brown et al. 2011), specific actions of individual anesthetics can occur both independently and along with a common mechanism that leads to the state of unconsciousness. Only a few proposals that explain a common general mechanism for the action of all the general anesthetics at the cellular molecular level were made and are described below.

## Lipid membrane and other hypotheses

The membrane hypothesis is generally stated as the Mayer (1899) and Overton (1901) rule based on which the potency of an anesthetic is increased in proportion to its partition coefficient (concentration ratio) between olive oil and water (hydrophobic solubility). An experimental demonstration of this showed a correlation coefficient of 0.997 (Firestone et al. 1986). This correlation among inhaled anesthetics with potencies ranging over 100,000-folds has been viewed as one of the most powerful correlations in biological systems (Halsey 1992). Investigations show that anesthetics dissolve in the hydrophobic region of the membrane causing this region to expand, which led to the critical volume hypothesis (Miller et al. 1972a, b). The membrane expansion by anesthetics was confirmed on erythrocyte membranes (Roth and Seeman 1972). Specific mechanisms related to the spontaneous curvature of lipid membranes were also proposed (Gruner and Shyamsunder 1991; Lenaz et al. 1978). Modification of the lateral phase separation properties of the membranes with a resulting inability of membrane proteins to change conformation or undergo insertion into the lipid membrane were proposed (Trudell 1977; Cantor 1997; Brown et al. 2011). Another proposal was the multi-site expansion hypothesis (Halsey et al. 1978). Based on the binding of general anesthetics to the hydrophobic sites, several other hypotheses were also generated (Rosenberg et al. 1975; Haydon et al. 1977; Vanderkooi 1977). In short, lipid membranes have been at the central-point of the mechanism of action of different anesthetics.

The information-processing hypothesis of anesthetic mechanism (Flohr 1995) was based on the premise that glutamatergic NMDA receptors affect Hebbian plasticity. This was supported by the finding that anesthetics that are GABA receptor agonists can have an inhibitory effect on the NMDA receptor-mediated actions. Even though anesthetics are thought to disrupt higher-order cortical information integration (Hudetz 2012), a fully reversible mechanism of anesthesia during withdrawal of anesthetics and by application of pressure has not yet been discovered.

## Basic structure of the nervous system

The sensory identity of the first-person internal sensation of various higher brain functions, to which only the owner of the nervous system has access, requires a neuronal circuit mechanism explaining the induction of internal sensory elements. The circuitry with this function is expected to be connected to the motor neurons for behavioral motor activity. What cellular mechanism can impart internal sensory elements to the system? Consciousness being generated autonomously within the system is dependent on the specific frequency of surface or extracellular recorded oscillatory patterns of potentials in the cortex. Therefore, mechanisms that induce internal sensations are related to one or more vector components responsible for oscillating potentials. Therefore, it is necessary to fully understand both the synaptic connections between the vertically oriented neuronal orders in the cortex that can provide the vertical component and potential mechanisms that can provide the horizontal component for the oscillating potentials.

The nervous system has synaptically connected neurons with a widely varying number of input and output terminals. The number of input terminals (postsynaptic terminals or postsynapses or dendritic spines) to a neuron ranges from approximately 5600 (monkey visual) to 60,000 (monkey motor) (Cragg 1967). A synapse is the junction between an output terminal (presynaptic terminal) and an input terminal. Excitatory neurons and their connections are taken as the primary circuit elements that are regulated by inhibitory interneurons. The inputs arrive at the postsynapses in the form of excitatory postsynaptic potentials (EPSPs). Spikes of potentials are seen at different locations on the neuronal processes: dendrites (dendritic spikes or regenerative potentials), the axonal hillock (axonal spikes or action potentials) and the cell body (somatic spikes or neuronal firing). Action potential generated at the axonal hillock area of the neurons is essential for the propagation of activity to higher neuronal orders. An excitatory neuron fires when nearly 40 EPSPs summate spatially (Palmer et al. 2014), or even less than 40 EPSPs summate temporally close to the axon hillock, indicating that large numbers of EPSPs in excess or less than the threshold values are not used for eliciting an action potential. Therefore, their evolutionary preservation is not yet known. Similarly, the functional significance of dendritic spikes is also not yet known. The contribution of EPSPs generated at the apical dendrites towards somatic spikes is minimal, making it essential to make an inquiry to understand their functional significance. In these contexts, all the EPSPs generated at various locations are examined for whether they contribute to the generation of internal sensory elements for higher brain functions.

## Framework of consciousness from semblance formation

The semblance hypothesis was developed to explain the basic mechanism of the formation of first-person internal sensations of various higher brain functions to which only the owner of the nervous system has access. Continuous quantal release of the neurotransmitter takes place all the time from single vesicles in the presynaptic terminals to the synaptic cleft. In addition, the arrival of an action potential at the presynaptic terminal triggers the release of a volley of neurotransmitter into the synaptic cleft. In both these conditions, the postsynaptic terminal develops potentials as a result of the release of the neurotransmitter from its presynaptic terminal. Activating the postsynaptic terminal in the absence of the release of the neurotransmitter from the presynaptic terminal was hypothesized to induce units of internal sensations generating semblance of the arrival of activity from the presynaptic terminal.

**Fig. 1:**
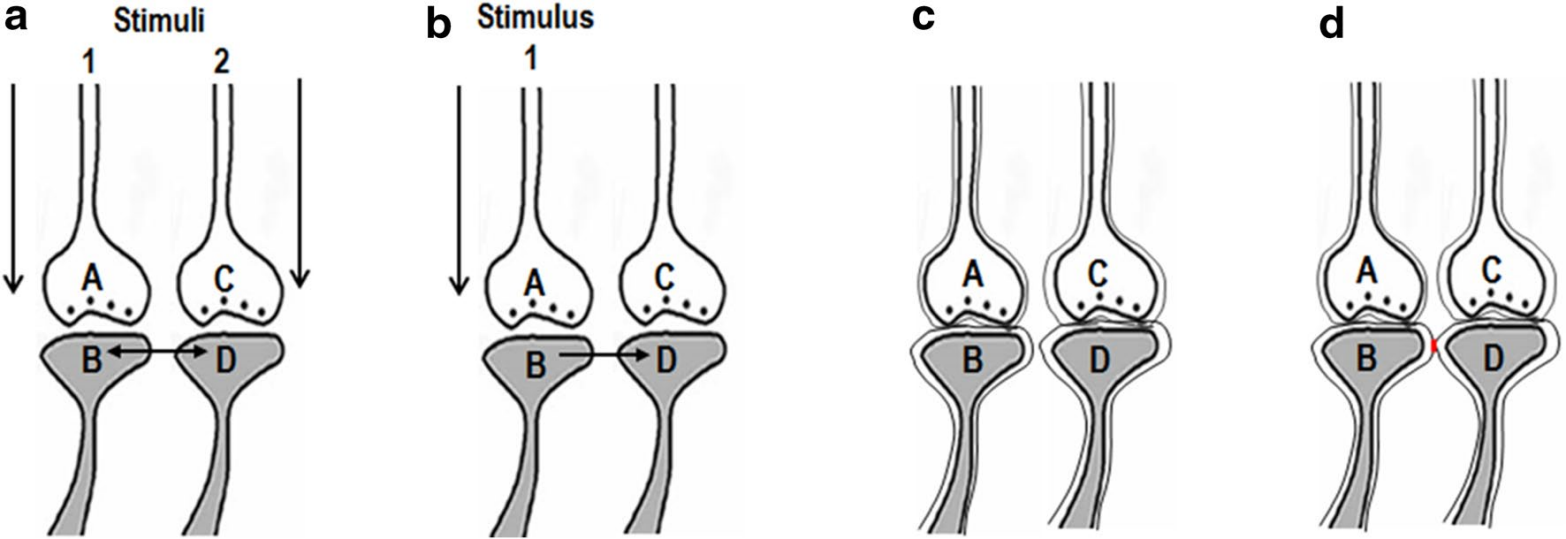
Formation and reactivation of inter-postsynaptic functional LINKs. **a** The illustration shows functional LINK formation between two postsynaptic membranes (postsynapses or dendritic spines) *B* and *D* when they are simultaneously activated when two stimuli are associated. The functional LINK is reversible, stabilizable and its formation is a function of the simultaneous activation of postsynapses *B* and *D*. *A* and *C* are corresponding presynaptic terminals. **b** At a later time, when one of the stimuli arrives at postsynapse *B* through synapse *A*–*B*, functional LINK *B*–*D* is re-activated, resulting in the activation of postsynaptic membrane *D*. This induces a unit of internal sensation of activity arriving from presynaptic terminal *C*. The reactivation of the functional LINK is a function of arrival of activity at one of the postsynaptic terminals. **c** Two abutted synapses are shown with their presynaptic and postsynaptic terminal membranes in lipid bilayers. Note that the postsynaptic membranes are separated by extracellular matrix space. **d** The formed inter-postsynaptic functional LINK is shown in *red*. Both direct membrane contact by excluding inter-membrane hydrophilic region and reversible partial membrane hemifusion are common mechanisms (Figure modified from Vadakkan 2010)

**Fig. 2:**
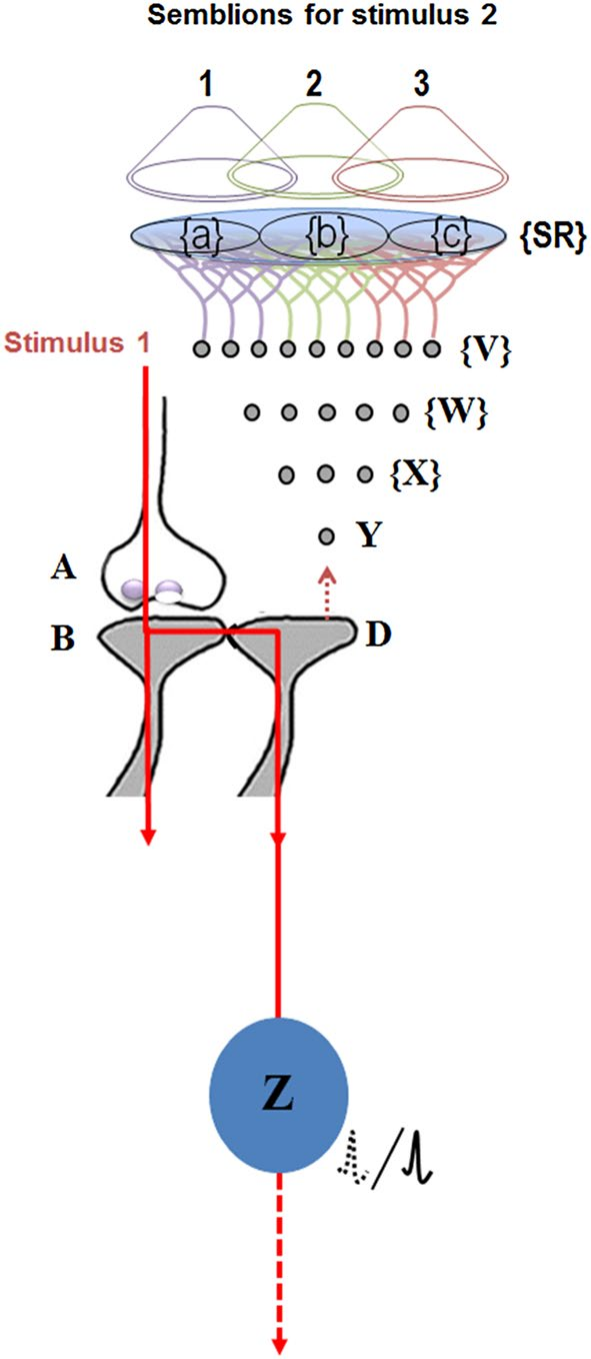
Reactivation of an inter-postsynaptic functional LINK that induces the formation of units of internal sensation. An action potential arriving at presynaptic terminal *A* activates synapse *A*–*B* and reactivates inter-postsynaptic functional LINK between postsynapses *B* and *D*. When postsynapse *D* is activated in the absence of arrival of activity from its presynapse (not shown), a semblance of arrival of activity from its presynapse occurs. The sensory equivalent of the semblance (sensory hallucinations) can be extrapolated from examining the packets of minimum sensory stimuli capable of stimulating postsynapse *D*. The sensory identity of the semblance of activity occurring at postsynapse *D* consists of inputs from neuron *Y*. Neuron *Y* is normally activated by inputs from a set of lower order neurons {*X*}. Continuing this extrapolation towards the sensory level identifies a set of sensory receptors {*SR*}. {*a*}, {*b*} and {*c*} are subsets of {*SR*} and are capable of independently activating postsynapse *D*. Hypothetical packets of sensory stimuli activating sensory receptor sets {*a*}, {*b*} and {*c*} are called semblions *1*, *2* and *3* respectively. Activation of postsynapse *D* through inter-postsynaptic functional LINK *B*–*D* by the cue stimulus can lead to the virtual internal sensation of semblions either *1*, or *2*, or *3* or their integral or their overlapping region. Cue stimulus-induced activation of postsynapse *D* reaches the soma of its neuron *Z*. If neuron *Z* already receives baseline summated EPSP short of one EPSP to trigger an action potential, then the additional EPSP arriving through inter-postsynaptic functional LINK *B*–*D* and through postsynapse *D* can add to the sub-threshold EPSP and fire neuron *Z*, resulting in latter’s concurrent activation during the formation of internal sensation (Figure modified from Vadakkan 2010)

Potentials arriving at the postsynaptic terminal through a LINK (the word “link” is highlighted to emphasize its importance) from the neighboring postsynaptic terminal can evoke units of internal sensations eliciting the semblance of the arrival of activity from the presynaptic terminal. An inter-postsynaptic functional LINK is expected to form between the abutted postsynaptic locations as a function of the simultaneous arrival of activity from two different sensory inputs during associative learning between two stimuli (Fig. 1a). The reactivation of the LINK occurs as a function of the arrival of activity from the one of the associatively learned stimuli through the inter-postsynaptic functional LINK to the inter-LINKed postsynaptic terminal (Fig. 1b). Since lipid bilayers of different postsynaptic terminals (Fig. 1c) abut each other with a negligible extracellular matrix volume, as visualized in electron microscopic pictures, an interaction between their outer layers is expected to occur (Fig. 1d). The sensory identity of the semblance of the second stimulus formed at a postsynaptic terminal by the reactivation of the inter-postsynaptic functional LINK by the arrival of activity from the first stimulus and how it can be derived are explained in Fig. 2.

Phenomenal properties of consciousness such as subjectivity and intentionality can be viewed only from a first-person perspective (Velmans 1991). Consciousness is seen as a binding process by which different internal sensations evoked by an item are associated in the nervous system similar to that taking place during associative learning (Vadakkan 2010). Spontaneous potentials induced during dendritic spikes and the continuous arrival of background sensory stimuli from inside the body and environment can contribute to the surface or extracellular recorded oscillating potentials. During these, inter-LINKed postsynapses are reactivated and concurrently induce semblances at large number of postsynaptic terminals. The apical area in the cortical layer 1 where apical tufts from all cortical neuronal orders anchor is a potential area where inter-postsynaptic LINKs are expected to be densely present. Local dendritic spikes observed in in vitro experiments (Regehr et al. 1993; Polsky et al. 2009) were recently shown to be present in in vivo by different studies (Palmer et al. 2012; Sheffield and Dombeck 2015; Cichon and Gan 2015). The potentials from these dendritic spikes from the apical tufts degrade significantly as they reach the soma, making their contribution to somatic spike often insignificant. In this context, their evolutionary preservation implies their yet-unknown functional role. In this context, the sensory elements imparted by spontaneous events like dendritic spikes can be equated to the semblances described in Fig. 2 and are examined for their contribution to the internal sensation of consciousness. Semblance induced at the postsynaptic terminal by its stimulation, in the absence of the arrival of activity from its presynaptic terminal, can be viewed as a mechanism evoking internal sensations. From a large number of findings that localized stimulation of the extracellular matrix space (ECM) at specific sensory cortices induces related sensory hallucinations (Selimbeyoglu and Parvizi 2010), it can be inferred that units of internal sensation can be induced by spontaneous activation of certain neuronal processes.

**Fig. 3:**
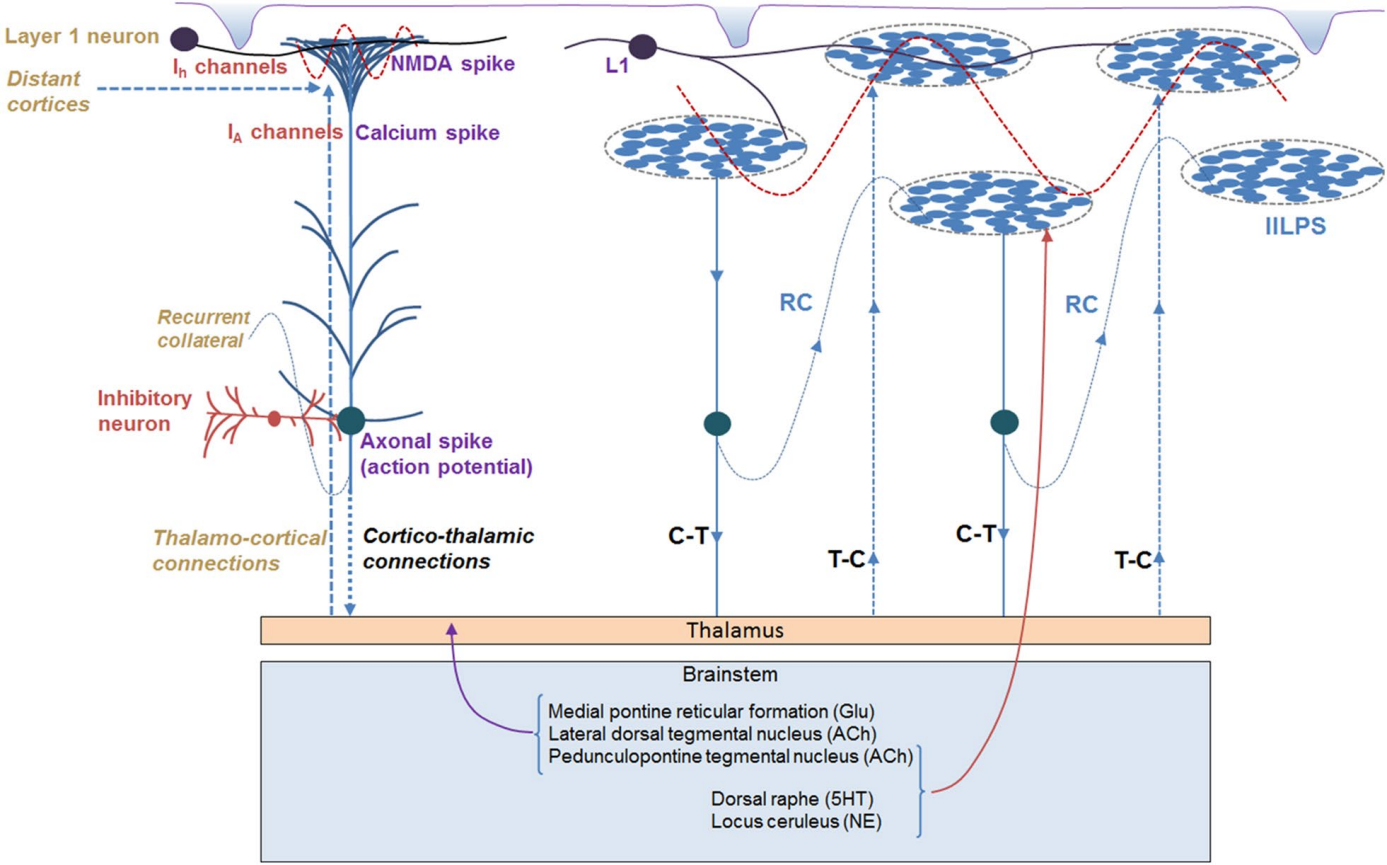
Sources of potentials that contribute to the horizontal and vertical components of the oscillating potentials. *Upper left side* A cortical pyramidal neuron with different locations of spike generation. The source of surface-recorded electro-encephalogram (EEG) waveforms is likely to have significant contributions from the NMDA spikes from the apical tufts since their magnitude is higher than that of the somatic spikes (neuronal firing) and they occur close to the pial surface. *Upper right side* Five islets of inter-LINKed postsynaptic terminals (IILPS) are shown that represent the abundance of dendritic spines in this area that permits several postsynapses to get inter-LINKed both by innate and acquired mechanisms. The islets are expected to be connected with each other through recurrent collaterals, layer 1 cortical neurons and cortico-thalamo-cortical pathways. This pattern of arrangement will provide a mechanism for long-range synchronization that is being recorded as EEG waveforms. *Bottom* The role of both thalamus and brain stem inputs in maintaining the frequency of oscillations in the cortex. Various nuclei in the brain stem that provide inputs to both thalamus and cortex are shown (neurotransmitters are given in *brackets*). Cortico-thalamo-cortical pathway maintains a significant role in controlling the oscillating potentials in the cortex. Pontine reticular activating system sends glutamatergic inputs to the thalamus potentially regulating the oscillating potentials in the cortex. *RC* recurrent collateral, *C-T* cortico-thalamic pathway, *T-C* thalamo-cortical pathway, *L1* layer 1 cortical neuron, *IILPS* islet of inter-LINKed postsynapses, *Glu* glutamate, *ACh* acetyl choline, *5HT* 5-hydroxy tryptamine (serotonin), *NE* nor-epinephrine

The dendritic spikes indicate that potentials are spontaneously generated at several postsynaptic terminals. The resistive properties of the long thin spine necks result in large potentials at the spine heads (postsynaptic terminal). There are two findings that need a matching explanation. First, the postsynaptic terminals are abutted to each other at the apical tuft area. The second one is the presence of surface or extracellular recorded oscillatory potentials. Since the oscillating potentials require a mechanism for their horizontal components, an innate mechanism whereby several postsynaptic terminals form islets of inter-LINKed postsynapses (Fig. 3) is expected to occur at areas of the cortex where postsynaptic terminals of different neurons abut each other. The changes in the horizontal component can then be examined for the observed changes in the frequency of oscillations during various conditions.

Pulvinar, mediodorsal, intralaminar and midline nuclei of the thalamus receive major inputs from cortical layers 5 and 6 and project back to the cerebral cortex to form cortico-thalamo-cortical pathways (Guillery 1995; Sherman and Guillery 2002; Theyel et al. 2010) that can regulate the oscillating potentials. In addition, the horizontal spread of activity through horizontally located processes of the layer 1 neurons, recurrent collaterals and inhibitory interneurons (Palmer et al. 2012) are potential factors that regulate oscillations of potentials. Awareness of the self and the environment requires the functioning of the ascending reticular activating system (ARAS) originating from the reticular formation (RF) in the brain stem and relayed through the intralaminar nucleus of the thalamus to the cerebral cortex (Kinomura et al. 1996; Edlow et al. 2012; Yeo et al. 2013; Saalmann 2014). In addition to the brain stem nuclei including the locus coeruleus, dorsal raphe, median raphe, pedunculopontine nucleus, and parabrachial nucleus, the ARAS also includes non-specific thalamic nuclei, the hypothalamus and the basal forebrain (Aston-Jones et al. 2001; Parvizi and Damasio 2003).

**Fig. 4:**
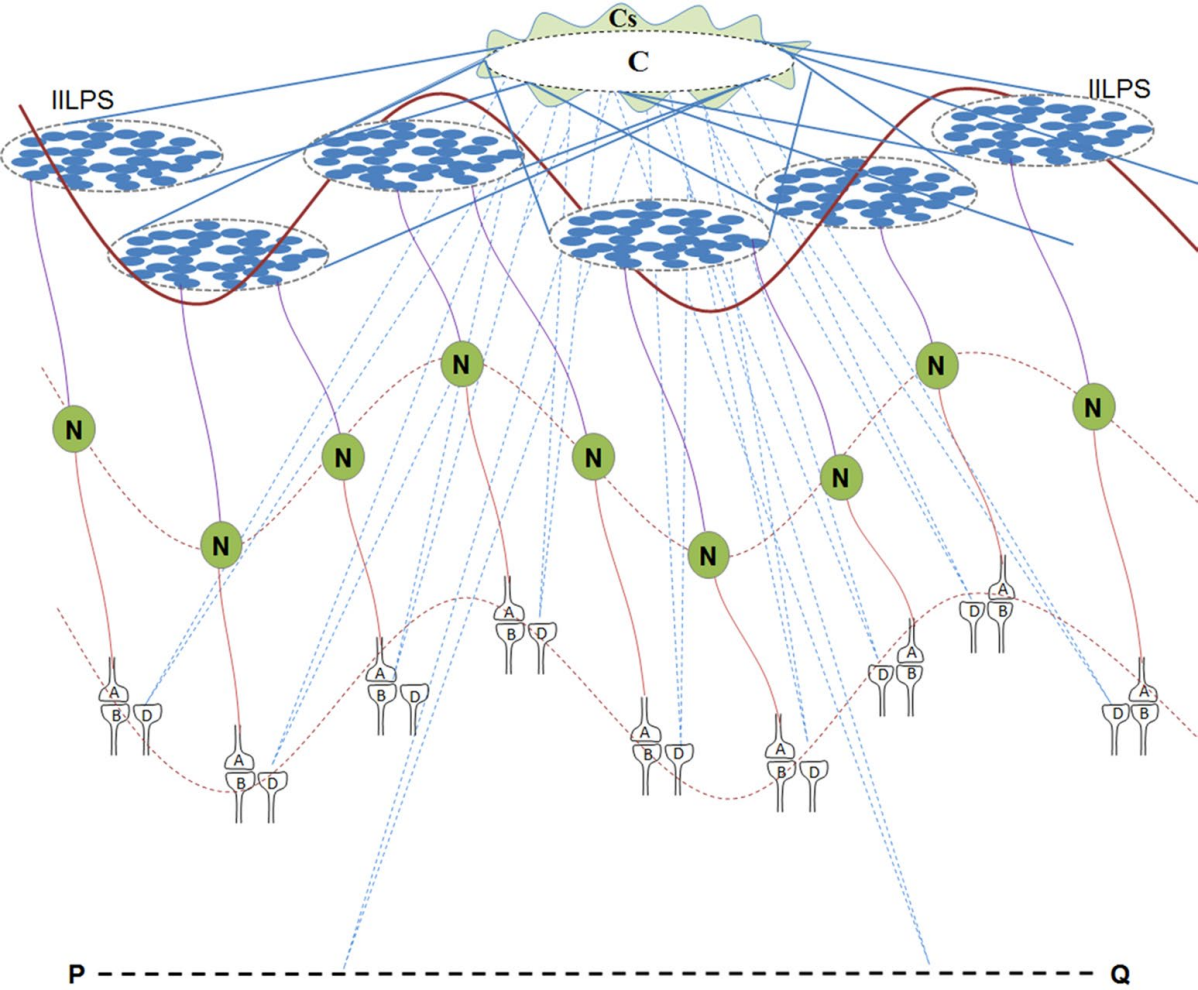
Formation of C-semblance for consciousness and the role of oscillating potentials. Spontaneous activity occurring during events such as dendritic spikes occurring at the islets of inter-LINKed postsynapses (IILPS) can lead to semblance at all the postsynapses that are activated thorough the LINKs (in the absence of arrival of activity from their presynaptic terminals). In addition, reactivation of large number of scattered single inter-postsynaptic functional LINKs (*B*–*D*) within the cortices also induce semblances. Background sensory stimuli both from within the body (respiration and heart beat) and from the environment reactivate several inter-postsynaptic functional LINKs and also induce semblances. The net result of all the semblances induced at the postsynapses lead to C-semblance for consciousness. C-semblance is a function of (a) the reactivated inter-postsynaptic LINKs that in turn is a function of existing innate inter-postsynaptic LINKs, (b) some of the acquired inter-postsynaptic LINKs induced by associative learning during life that are reactivated during oscillation of potentials and (c) the complexity of the nervous system of a given species. The synaptic transmission between vertically oriented neuronal orders provides vertical component and the lateral spared of potentials among the postsynapses during dendritic spikes provide horizontal component that lead to surface or extracellular recorded oscillating potentials. These oscillating potentials eventually give rise to neuronal firing (somatic spikes) in an oscillating manner (shown by a *wave-form*). Additional factors that provide components for oscillations include recurrent collaterals, laterally connected layer 1 neurons, cortico-thalamo-cortical connections and activity arriving from the thalamus (in response to background sensory stimuli) and the brain stem (connections with respiratory drive). When frequency of oscillating potentials changes, the nature of consciousness will change (example, in sleep). *C* C-semblance, *Cs* subjective changes to C-semblance due to contributions of semblances induced through reactivation of inter-postsynaptic functional LINKs formed by different associative learning events during life. *P*–*Q* Represents background sensory inputs. *Wave shape* Represents oscillating pontentials. *A* Presynaptic terminal where inputs from oscillatory neuronal activity arrives. *B* Postsynaptic terminal of the synapse *A*–*B*. *D* Postsynaptic terminal which is functionally LINKed to the postsynaptic terminal *B*. *B*–*D* Inter-postsynaptic functional LINK. Corresponding presynaptic terminal of postsynapse *D* is not shown. *N* Neurons that are firing. *P*–*Q* Represents background sensory inputs arriving at the nervous system either from the environment or from the body such as respiration and heartbeat. *IILPS* Islet of inter-LINKed postsynapses (Figure modified from Vadakkan 2010)

Continuous formation of semblances occurs during dendritic spikes and other spontaneous activations of the postsynaptic terminals (in the absence of the arrival of activity at the presynaptic terminals). The composition of all the background semblances induced in a modular fashion at different cortices in the resting state leads to the formation of C-semblance (the net semblance for consciousness) responsible for consciousness (Fig. 4) (Vadakkan 2010). Since lower forms of animals show intentionality to carry out basic motor behaviors for feeding and reproduction, which are essential for maintaining the species, a robust circuit property is expected to be present in all the nervous systems that induce internal sensations for maintaining states equivalent to awareness. Consciousness is strongly associated with the specific frequency of the surface or extracellular recorded oscillating potentials. The dendritic spikes that involve a large number of synapses at one location are likely to activate postsynapses within islets of inter-LINKed postsynapses providing the required horizontal component for oscillating potentials for C-semblance. Synaptic transmission between the vertically oriented neuronal orders in the cortex provides the vertical component for the oscillations. C-semblance act as a background or buffer during active computations—for example, during the matching process of the formed internal sensation of retrieved memory with that of the learned item.

The inputs from both the thalamus and those that directly reach the cortex are expected to maintain an appropriate frequency of the oscillating potentials. Any disturbance in the inputs from the thalamus or the reticular formation can disturb consciousness, which is reflected in the frequency of the waveform of potentials recorded from surface or extracellular electrodes. This indicates that the conformation of net C-semblance occurring through the composition of units of internal sensations for the systems property of consciousness in the cortex is dependent on the frequency of oscillating potentials. Factors that regulate the optimal frequency of oscillatory potentials determine the optimal conformation of the C-semblance for consciousness. The computational process of composing the semblances that provides optimal conformation of C-semblance (Vadakkan 2010) requires further investigations. Back propagation of somatic action potentials from individual pyramidal neurons is unlikely to reach towards the apical dendritic spines and disturb the latter’s internal sensory contributions to consciousness. This is supported by both theoretical estimations (Behabadi and Mel 2014) and by the failure of back propagation of action potentials to reach the distal dendrites of the apical tuft using calcium imaging experiments (Schiller et al. 1995), even though they can be recorded at the level in the dendrite at which patch clamping can be carried out (Stuart et al. 1997).

## Horizontal component of oscillating potentials

Alteration in consciousness is associated with a reduction in the frequency of surface oscillating potentials; this indicates that either the contribution of the horizontal component increases or that of the vertical component decreases. One of the potential sources of surface recorded potentials is the spontaneous activity at the dendritic spines (postsynapses) that lead to dendritic spikes at the apical tuft area of the cortices. Dendritic spikes that spread to other postsynapses through inter-postsynaptic functional LINKs are a candidate mechanism for the horizontal component of oscillating potentials. An innate mechanism that inter-LINKs several postsynapses is a possible method for the horizontal spread of potentials. Evidence that the discontinuous electroencephalogram (EEG) waveforms in very premature infants (Selton et al. 2000) eventually get connected from their lateral ends indicates that lateral connectivity is an essential component for oscillating surface recorded potentials and is produced through an innate mechanism. When the frequency of oscillating potentials is reduced both during sleep (Alkire et al. 2008) and anesthesia (Sanchez-Vives and McCormick 2000), consciousness is altered. Thalamo-cortical oscillating potentials that differ during sleep and awake states (Steriade et al. 1993) have a significant role in maintaining oscillating potentials in the cortex recorded from the surface, in a plane parallel to the pia mater. One of the consequences of oscillating potentials recorded in the apical tuft area is the firing of several downstream neurons in an oscillating manner.

## Inter-postsynaptic functional LINK

The duration of existence of an inter-postsynaptic LINK correlates with the type of internal sensations being induced during different higher brain functions. Examining all the different changes can help with understanding a possible mechanism of action for anesthetics. For explaining the internal sensation of perception, inter-postsynaptic functional LINKs should be either preexisting or have a very rapid turnover of the formation and reversal steps. The duration of persistence of inter-postsynaptic functional LINKs for generating internal sensations during working, short-term and long-term memories increases proportionate to the duration of the persistence of memory. In the case of consciousness, the background level of the sub-conscious state during sleep should operate via a well preserved innate mechanism through stable inter-postsynaptic functional LINKs. The changes in the level of consciousness during different stages of sleep are expected to depend on the addition of a rapidly reversible mechanism. The latter is expected to take place in different proportions corresponding to different stages of sleep that lead to corresponding deviations in the conformation of C-semblance from that in the waking state. The semblances that are induced during arousal from sleep should be occurring through reversible mechanisms. From examining different mechanisms of the formation and reactivation of inter-postsynaptic functional LINKs, the effect of anesthetics on a reversible mechanism can be understood.

**Fig. 5:**
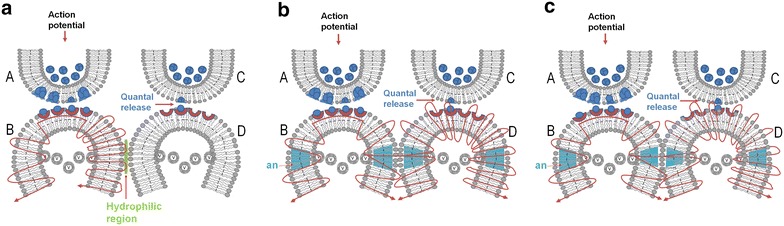
Formation of different types of reversible inter-postsynaptic functional LINKs. **a** Two abutted synapses *A*–*B* and *C*–*D*. Presynaptic terminals *A* and *C* are shown with synaptic vesicles (in *blue color*). Action potential arrives at presynaptic terminal A releasing a volley of neurotransmitters from many synaptic vesicles inducing an excitatory postsynaptic potential (EPSP) at postsynaptic terminal *B*. The waveform represents the direction towards which the EPSP propagates. From the presynaptic terminal *C*, one vesicle is shown to release its contents to the synaptic cleft. This quantal release is a continuous process (even during rest) providing very small potentials to postsynaptic membrane *D*. Postsynaptic terminals *B* and *D* have membrane-bound vesicles marked *V* inside them. These vesicles contain glutamate receptor subtype 1 (GluA1). Activity arriving at the synapse can lead to exocytosis of GluA1 receptor-subunits and expansion of the postsynaptic membrane. During exocytosis, the vesicle membrane is added to the postsynaptic membrane at locations of exocytosis making this region of the membrane highly re-organisable. This matches with the location where α-amino-3-hydroxy-5-methyl-4-isoxazole-propionic acid (AMPA) receptor subunits were shown to concentrate at the extra-synaptic locations extending at least 25 nm beyond the synaptic specialization (Jacob and Weinberg 2014). Note the presence of a hydrophilic region separating postsynaptic terminals *B* and *D*. When action potential arrives at the presynaptic terminal, it activates synapse *A*–*B* and an EPSP is induced at postsynaptic terminal *B*. The hydrophilic region prevents any type of interaction between postsynapses *B* and *D*. Very high energy is required for excluding the inter-postsynaptic hydrophilic region (Martens and McMahon 2008). **b** Diagram showing the effect of lipophilic anesthetic molecule on the membranes. Incorporation of the hydrophobic anesthetic molecule to the lipid membrane especially at the re-organisable areas that lead to membrane expansion at these locations can provide sufficient energy to exclude the inter-postsynaptic hydrophilic region allowing close contact between the postsynaptic membranes at this region. Action potential arriving at synapse *A*–*B* reactivates the inter-postsynaptic functional LINK formed by close inter-postsynaptic contact and spreads to postsynaptic terminal *D*. *an* membrane segment marked in Turkish blue shows area where membrane reorganization occurs and anesthetic molecules lead to membrane expansion. Formation of large number of non-specific inter-postsynaptic functional LINKs disturbs the net C-semblance formation explained in Fig. 4 leading to loss of consciousness. When the anesthetic molecule is removed, the process reverses back. **c** Diagram showing formation of a partial inter-postsynaptic membrane hemifusion following anesthesia. Note the interaction between the outer layers of membranes of the postsynaptic terminals. Depending on the lipid membrane composition and the type and concentration of anesthetics, the process of close contact between the membranes described in above section (**b**) can get converted to a partial hemifusion state. The process can even advance to a reversible complete hemifusion state as described in Fig. 8b depending on several factors. When the anesthetic molecule is removed, the hemifusion reverses back

Different candidate mechanisms for the inter-postsynaptic functional LINK include direct membrane contact excluding the inter-membrane hydrophilic region, reversible partial and complete membrane hemifusion (Vadakkan 2013) and a still unknown mechanism operating through the ECM. The hydration repulsive force between two artificial lipid membranes maintains a distance of nearly 2 nm between the membranes (Markin et al. 1984). Diminishing the inter-membrane hydration repulsion is one of the methods of initiating membrane contact (Rand and Parsegian 1989). The direct membrane contact excluding the inter-membrane hydrophilic region is an ideal mechanism due to its rapid reversibility. Membrane dynamics at the postsynaptic membrane very close to the synapse is a favorable location for achieving direct membrane contact by excluding the inter-membrane hydrophilic region (Fig. 5a, b).

Long-term potentiation (LTP), an electrophysiological experimental finding that correlates with the surrogate behavioral motor activity indicative of the formation of the internal sensation of retrieved memories, helps us understand the probable mechanism of inter-postsynaptic functional LINK (Vadakkan 2013). Several studies have shown that dendritic spines (postsynapses) enlarge following LTP induction (Buchs and Muller 1996; Maletic-Savatic et al. 1999). Since extracellular matrix between the postsynapses is negligible, especially at the locations of the convergence of inputs, the dendritic spine enlargement increases the probability of getting them abut to each other. Since LTP was attenuated by the injection of synaptosomal-associated protein (SNAP) inhibitors, which inhibit membrane fusion, into the cell body of CA1 neuron (Lledo et al. 1998), some stages in the membrane fusion process are expected to be involved in the induction of LTP (Vadakkan 2013). The ideal candidate mechanism is reversible membrane hemifusion, which is the initial stage during the fusion process commonly seen in biological systems (Melikyan and Chernomordik 1997; Kozlov et al. 2010).

Postsynaptic membranes at the lateral edges can get constantly exchanged with vesicle membrane lipid bilayer segments during exocytosis and endocytosis of the GluA1 subunits of one type of the glutamate receptors called α-amino-3-hydroxy-5-methyl-4-isoxazole-propionic acid (AMPA) receptors (Passafaro et al. 2001). The observation of an increase in the volume of dendritic spines even before the accumulation of GluA1AMPA receptor subunits (Kopec et al. 2006) is also a possible mechanism that augments membrane hemifusion. Vesicle membranes of GluA1AMPA receptor-subunit-containing endosomes (Park et al. 2004) are primarily involved in the postsynaptic membrane reorganization. The biochemical conditions and membrane lipid composition can determine conversion of direct contact between the membranes to reversible membrane hemifusion. GluA1AMPA receptor subunits have been reported to enter the plasma membrane of dendrites in response to intense synaptic activity (Shi et al. 1999). It has recently been found that GluA1AMPA receptor subunits concentrate towards the extra-synaptic locations extending at least 25 nm beyond the synaptic specialization (Jacob and Weinberg 2014), making this area match the locations where postsynaptic membranes can hemifuse.

Studies using artificial membranes have shown that the membrane fusion process (of which membrane hemifusion is the initial step) has to overcome a high energy barrier (Cohen and Melikyan 2004; Martens and McMahon 2008) and therefore the locations of hemifusion are expected to be restricted to very small areas of approximately 10 nm^2^ similar to the findings in studies using artificial membranes (Leikin et al. 1987). Dedicated studies to examine inter-postsynaptic membrane hemifusion have not been undertaken due to the need for high-resolution microscopic techniques for live imaging and the requirement of high resolution electron microscopic imaging to observe areas as small as 10 nm^2^. Electron microscopic examination using the best available tissue preparation and resolution methods has shown a pair of abutted postsynaptic membranes with half the number (two) of expected (four) lipid membrane layers (Burette et al. 2012). In addition to changes that can accompany AMPA receptor expression (Matsuzaki et al. 2001), enlargement of the dendritic spines by dopamine-induced mechanisms (Yagishita et al. 2014) can promote membrane hemifusion in the context of associative learning. Inter-postsynaptic functional LINKs are formed at specific locations for the formation of internal sensations of different higher brain functions. However, since anesthetics bind to the lipid membranes non-specifically, they can either block or result in excessive formation of non-specific inter-postsynaptic functional LINKs.

## Proposed mechanism of anesthetics

The maintenance of the background set of inter-postsynaptic membrane functional LINKs is essential for the specific conformation of the C-semblance for consciousness (Fig. 4). Any alteration in the conformation of the C-semblance can lead to loss of consciousness. The abutted postsynaptic membranes are anchored to the ECM through structural proteins. Extra-synaptic locations on the postsynaptic membranes extending at least 25 nm beyond the synaptic specialization where GluA1AMPA receptor subunits concentrate (77) are regions of continuous exocytosis and endocytosis. Since this process involves the addition and removal of lipid membrane segments, lateral aspects of postsynaptic membranes immediately outside the synapse is an area of dynamic membrane reorganization. Membranes at the resting state have a specific conformational energy. Different forces that keep the membranes separate include hydration, electrostatic and steric forces, whereas van der Waal’s forces bring two membranes together (Cevc 1987).

Associative learning produces changes at locations of convergence of stimulus inputs through enlargement of the postsynapses (dendritic spines) of synapses that abut each other. This change in spine geometry is found to be critical in AMPA receptor expression (Matsuzaki et al. 2001) and are expected to occur on the abutted postsynaptic membranes of those synapses that move close to each other. The finding that GluA1AMPA receptor subunits concentrate at the extra-synaptic locations, extending nearly 25 nm beyond the synaptic specialization (Jacob and Weinberg 2014), matches with the locations where postsynaptic membranes are expected to interact. A very large amount of pressure, nearly 10^9^ N/m^2^, is required to merge the outer leaflets of artificial membranes that are in contact (Markin et al. 1984). To minimize the work, the membrane hemifusion is expected to take place at a very small point of contact involving a minimum number of lipid molecules, making it a site-restricted process that is limited to areas as small as 10 nm^2^ (Leikin et al. 1987). In this respect, the localized area of membrane reorganization, 25 nm beyond the synaptic specialization where AMPA receptor subunit exocytosis takes place (Jacob and Weinberg 2014), is an ideal candidate location for inter-postsynaptic membrane interaction.

**Fig. 6:**
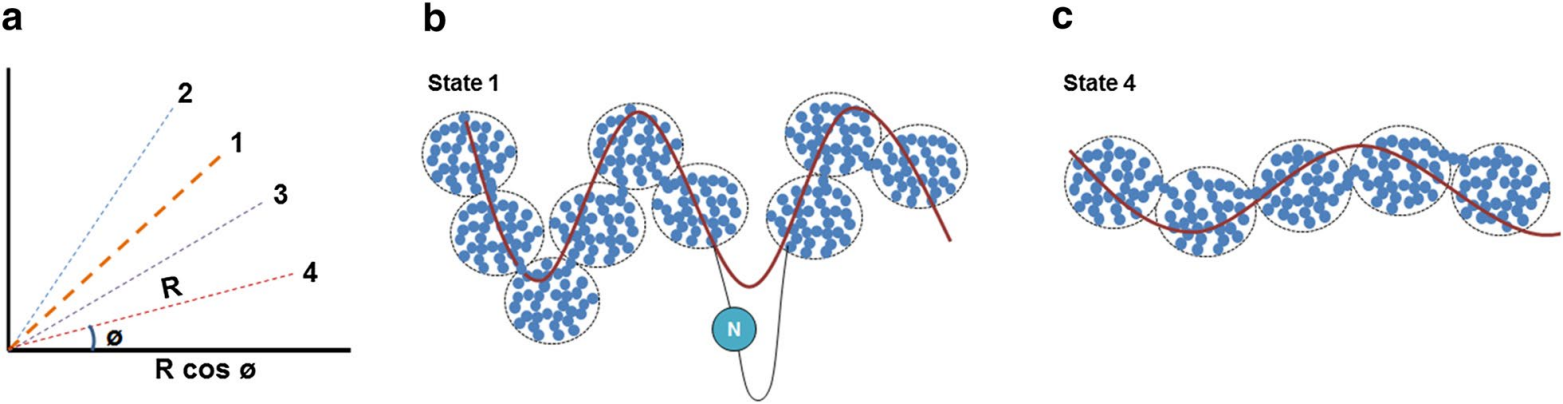
Increasing number of inter-LINKed postsynapses increases the horizontal component and reduces the frequency of oscillating potentials. **a** Graph showing the vector component of the oscillations. Four different states are marked. *1* Baseline state represented by equal vertical (synaptic) and horizontal (inter-postsynaptic functional LINKs) components. *2* As the horizontal component increases with increasing number of inter-LINKs between the islets of inter-LINKed postsynapses during the initial stage of anesthesia, more subthreshold neurons get activated increasing further vertical component. *3* Gradual increase in the number of inter-postsynaptic functional LINKs lead to increase in the horizontal component leading to gradual decrease in the frequency of oscillating potentials. *4* Represents phase 2 vegetative state in anesthesia where the frequency of oscillating potentials decreases further due to further increase in the horizontal component. **b** Oscillation of potentials during state 1 in the graph **a**, which is the normal baseline. Diagram showing islets of inter-LINKed postsynapses viewed as a cross-sectional view from above. For simplicity, the size of all the islets are drawn same. Spontaneous activity spreads across all the hemifused spines within that islet inducing semblances. *N* represents cortico-thalamic-cortical pathways and recurrent collaterals that contribute to the oscillating potentials. The frequency of oscillating potentials is determined by the horizontal component that depends on the inter-LINKs between the postsynapses horizontally. **c** Oscillation of potentials during state 4 in the graph **a**. Anesthetic molecules increase the number of inter-LINKed postsynapses and will inter-LINK several of the islets of already inter-LINKed postsynapses, increasing the magnitude of the horizontal component of the oscillating potentials. This reduces the measured frequency of these oscillations as shown by the *wave form* changes

The lipophilic anesthetic molecules are more likely to get partitioned inside the hydrophobic lipid phase in the regions of membrane reorganization at the postsynaptic membranes. The net result is the dehydration of the inter-membrane environment, which causes the abutted membranes to come into physical contact with each other (Fig. 5c). The spontaneous curvature induced by anesthetics arriving from the outside aqueous phase can contribute to asymmetry between the outer and inner leaflets of the lipid bilayer (Lipowsky 2014). The direct contact between the membranes that excludes the inter-membrane hydrophilic region is expected to be sufficient for the excitatory postsynaptic potential (EPSP) to spread from one postsynaptic membrane to the other. In this manner, anesthetics can induce a large number of non-specific inter-postsynaptic functional LINKs. It is observed that only reduced amounts of anesthetic agents are required for anesthesia in the presence of levodopa (Segal et al. 1990). Levodopa, known to cause the enlargement of dendritic spines (Meredith et al. 1995; Lee et al. 2006), supports the effect of dendritic spine enlargement in achieving direct contact between the spines as proposed by the present work. When islets of inter-LINKed postsynapses are inter-LINKed non-specifically, it will lead to alterations in the frequency of oscillating waveforms (Fig. 6) and conformation of C-semblance, resulting in changes in consciousness. The inter-postsynaptic functional LINKs induced by anesthetics can be readily reversed by removing the anesthetic agent.

## Stages of anesthesia and EEG waveforms

As the anesthetic dose is increased, the patients enter a state of paradoxical excitation characterized by euphoria or dysphoria, defensive or purposeless movements, and incoherent speech, along with an increase in beta activity (13–25 Hz) in the EEG. This state is termed paradoxical since the anesthetic, intended to induce unconsciousness, results in excitation (Brown et al. 2010). Initially, as the gradually increasing number of anesthetic molecules are bound to the lipid membranes, more inter-LINKs are formed between the existing islets of inter-LINKed postsynapses (Fig. 6a, b). Normally, oscillating potentials are expected to keep the output neurons in layer 5 of the motor cortex at a sub-threshold level of activation (Vadakkan 2013) that will enable them to fire upon the arrival of additional potentials. As anesthetics induce more inter-postsynaptic LINKs, this will lead to firing of several sub-threshold activated motor neurons in the motor cortex. This can explain the paradoxical excitation stage of anesthesia. As the depth of anesthesia is increased, EEG shows increasing slowness in the frequency of the waveforms. As more islets of inter-LINKed postsynapses get inter-LINKed, the magnitude of the horizontal component increases driving the frequency of oscillations to the lower side (Fig. 6c). This can explain the observed high gamma power during anesthesia (Murphy et al. 2011). The increase in the extracellular space after administration of ketamine/xylazine compared to the wake state (Xie et al. 2013) likely correlates with possible increased inter-postsynaptic membrane hemifusion.

When the anesthetic molecules get removed from the membranes after anesthesia is stopped, inter-postsynaptic LINKs at various stages revert back to their minimum energy state. This reverses the unconscious state induced by anesthesia back to the background conscious state. Propofol has one of the lowest potencies among the anesthetic agents since its octanol/water partition coefficient is one of the highest, obeying the Meyer–Overton rule (Tonner et al. 1992). This explains how once anesthesia is stopped, propofol can get displaced from the membranes very quickly, causing rapid reversal of the anesthetic effect and it is likely that propofol produces readily reversible inter-postsynaptic functional LINKs.

## Pressure reversal of the anesthetic effect

A large number of studies have confirmed that the general anesthesia induced by anesthetics is reversed by the application of pressure over an aquatic or terrestrial animal by increasing the pressure of water or air respectively within a closed container (Johnson and Flagler 1950; Johnson and Miller 1970; Lever et al. 1971; Miller 1974; Halsey and Wardley-Smith 1975; Kent et al. 1977; Beaver et al. 1977; Smith et al. 1984; Wann and Macdonald 1988; Daniels 2000; Chau et al. 2009). This leads to the natural question of how the pressure affects anesthetic action. Both hydrostatic pressure and gas phase pressure can be examined. Thermodynamically they act differently. While hydrostatic pressure exerts direct pressure, gas phase pressure changes solubility of the anesthetics in addition to its direct pressure effect. These observations lead to the following questions. How does externally applied pressure get transduced into the nervous system? Where is it getting transduced to? How does it produce the reversal of a general anesthetic-induced loss of consciousness? Knowledge of the route through which the externally applied pressure can transduce towards the membranes of neuronal processes and the mechanism that displace the membrane-bound anesthetic molecules is required.

First, an examination of the mechanism of hydrostatic pressure was carried out to understand the route through which externally applied hydrostatic pressure gets transduced into the extracellular matrix (ECM) space. It is known that pressure in the middle ear can get transmitted to the perilymph and then to the cerebrospinal fluid (CSF) (Martinez 1962), primarily through the cochlear aqueduct and secondarily through the endolymphatic duct and sac (Carlborg et al. 1982; Carlborg and Farmer 1983; Kishimoto et al. 1983). Recently, a new channel system called the glymphatic pathway (paravascular space) that directly connects CSF space to ECM space was discovered (Iliff et al. 2012). CSF space continues through these channels around the penetrating arteries and extends around the arterioles and around the capillaries. At the level of the capillaries, these channels are connected to the ECM space. After percolating through the ECM space, CSF flows to the paravenular space around the venular side of the capillaries and to the venous system. Nearly 5 μl of CSF are added, and the same volume is removed from nearly 150 ml of CSF volume during the period of one heartbeat, making the movement of CSF through the glymphatic pathway a convective flux (Kress et al. 2014). The very low flow of CSF provides a near-stable fluid compartment through which the applied pressure difference can get transduced to the ECM space. The neuronal processes are attached to the extracellular matrix through various structural proteins. At the locations close to the synapse, postsynaptic membranes undergo membrane reorganization as a result of exocytosis and endocytosis of AMPA receptor subunit-containing vesicles. This is viewed as an area where anesthetic-induced membrane changes can allow abutted postsynaptic membranes to come into close contact by excluding the hydrophilic region (dehydrating the inter-membrane region) in small areas.

**Fig. 7:**
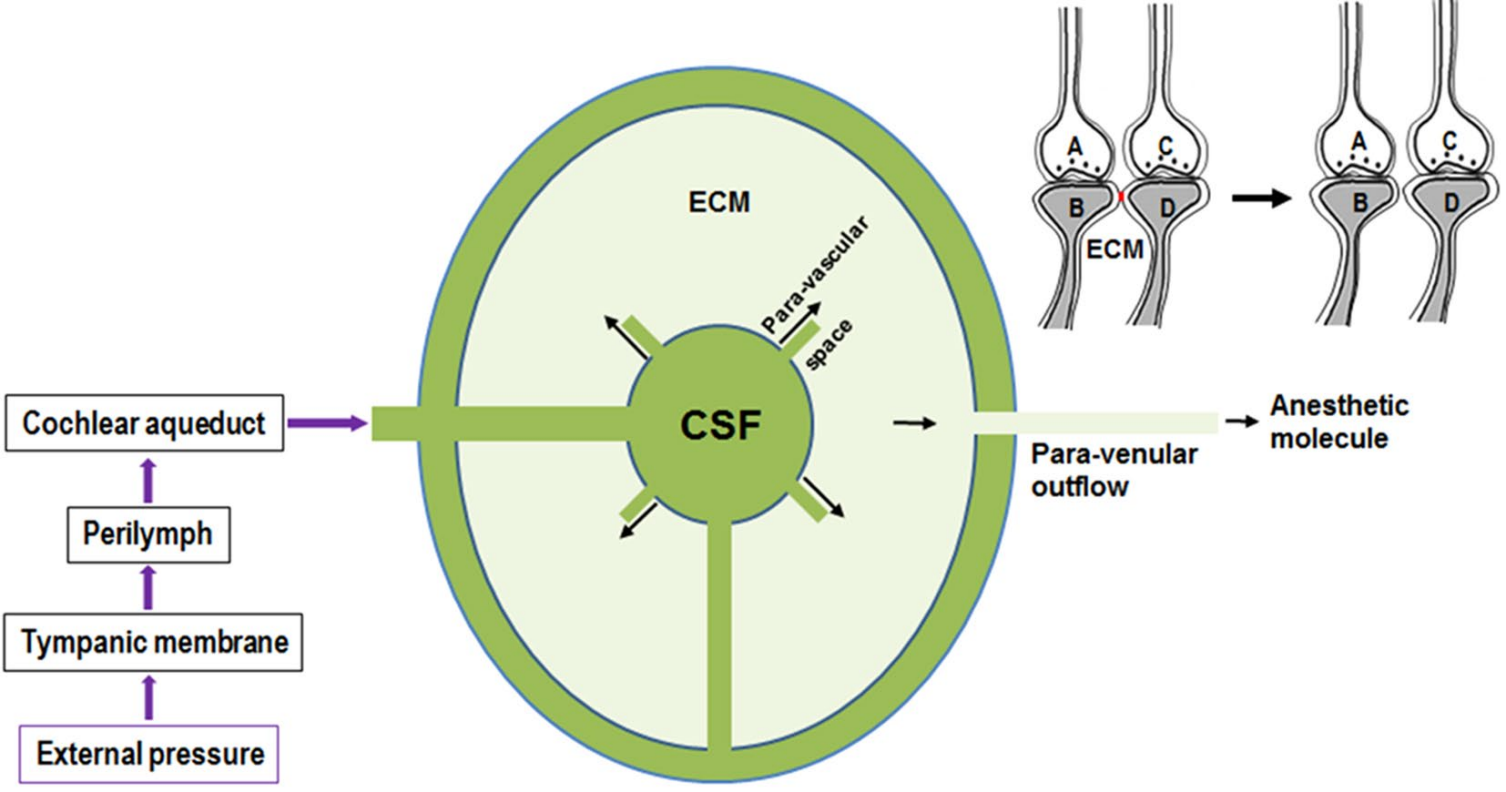
Route through which the externally applied pressure is transduced for the pressure reversal of anesthesia. Externally applied pressure get transduced to the cerebrospinal fluid (CSF) through the perilymph and cochlear aqueduct. This pressure gradient from CSF reaches the extracellular matrix (ECM) space through the glymphatic system (paravascular space). The pressure gradient gets transduced through the extracellular matrix space and result in displacement of the anesthetic molecules from the lipid membranes to the ECM and finally to the paravenular space and to the venous system. Both the close inter-postsynaptic membrane contacts and the reversible membrane hemifusions established in the presence of the anesthetics reverse back to the ground state. As the anesthetics get displaced, non-specific semblances induced through non-specific inter-postsynaptic functional LINKs will get proportionately reduced. This will bring back the normal conformation to the C-semblance as demonstrated in Fig. 4. *Top right* On the *left side* are two synapses with abutted postsynaptic membranes (dendritic spines) *B* and *D* in the presence of anesthetics forming an inter-postsynaptic functional LINK. Note the *red color* of the region of inter-postsynaptic functional LINK. On the *right side* is the state after pressure reversal of the inter-postsynaptic functional LINK. Inter-postsynaptic hydrophilic region forms again when anesthetic molecules are removed

How does the pressure gradient arriving at the ECM reverse the anesthetic-induced inter-postsynaptic functional LINKs? Based on Le Chatelier’s principle, when the pressure on a system at equilibrium is disturbed, the equilibrium position will shift in the direction necessary to reduce the pressure. One of the effects of increased pressure is the extrusion of anesthetic molecules from the lipid membranes to the ECM volume, and then these molecules get displaced through the paravenular space into the venous system. This in turn will reintroduce the hydrophilic region between the postsynaptic membranes, reversing the inter-postsynaptic functional LINKs induced by anesthetics (Fig. 7). Reversal of all the non-specific, inter-postsynaptic functional LINKs changing the C-semblance back into its normal conformation can explain the reversal of the unconscious state back to normal consciousness. In non-mammalian species such as freshwater shrimp (Simon et al. 1983) and nematodes (Eckenhoff and Yang 1994), pressure reversal of general anesthetics is not efficient, possibly due to the absence of the glymphatic pathway or due to some other structural variations.

The gas phase pressure mechanism is based on Dalton’s law of partial pressures that estimates the total pressure of a mixture of gases as the sum of the partial pressures of all the gases in the mixture. Gas phase pressure reversal can be made to occur by increasing the external pressure of the gas mixture, which will change the partial pressure of dissolved gases in the blood and eventually that of the extracellular matrix volume. During pressure reversal, partial pressure change reduces the solubility of anesthetic molecules and leads to their displacement from the lipid membranes into the ECM. In addition, the direct effect of external pressure on the middle ear that transmit through the perilymph, CSF, and paravascular space route as described for the effect of hydrostatic pressure can also occur. The displaced anesthetic molecules will then escape through the paravenular space into the venous system.

The effect of externally applied pressure in non-anesthetized humans (Bennett 1982) and animals (Brauer 1982) results in a well-studied phenomenon called high-pressure neurological syndrome (HPNS). In humans, it leads to tremors, psychomotor impairment, increase in theta activity in EEG and paradoxical hyper-excitability (Talpalar 2007). Pressure can lead to the displacement of CSF from the ECM through the paravenular space. This can lead to compression of the islets of inter-LINKed postsynapses. The lateral pressure over the postsynapses provides the energy required to remove the hydrophilic region between the postsynaptic membranes. The resulting increased number of non-specific inter-postsynaptic functional LINKs that are likely induced during HPNS can produce an effect similar to that of the excitement stage of anesthesia (described earlier) leading to increased motor activity.

## Effect of anesthetics on memory

The internal sensation of memory was explained in terms of semblances induced by the reactivation of inter-postsynaptic functional LINKs (26). The LINKs that are readily reversible explain working memory. Since they mimic the readily reversible action of anesthetics, the inter-postsynaptic functional LINKs induced during associative learning can be considered to take place via direct contact between specific postsynaptic membranes by excluding the inter-membrane hydrophilic region between them. Knowing that the natures of inter-postsynaptic functional LINKs formed to explain working memory (specific ones) and anesthetic action (non-specific ones) are similar provides an opportunity to examine the effect of anesthetics on memory. Low doses of anesthetics leave very short-term memory intact, such that patients can carry on a conversation and appear to be lucid (Wang and Orser 2011). As the anesthetic dose is increased, more non-specific inter-postsynaptic functional LINKs are induced, which will not allow sensory stimuli that are being associatively learned to converge at specific locations to form specific inter-postsynaptic functional LINKs that can be used for memory retrieval. Therefore, new learning will not become possible. Maintaining anesthetic-induced complete inter-postsynaptic hemifusions for long period of time increases the probability for their stabilization through the insertion of trans-membrane proteins that can lead to the prolonged inclusion of non-specific semblances from these postsynaptic locations.

A low dose of isoflurane [one-fifth required for immobilization (nearly 0.2 MAC)] suppresses learning and the explicit memory of verbal cues in healthy volunteers (Newton et al. 1990). Sub-sedative doses of isoflurane (0.3 %) and nitrous oxide (20 %) also impair immediate and delayed word recall (Zacny et al. 1994) by the same mechanism. Ketamine at sub-anesthetic doses in human volunteers reduces memory performance for explicit word recall (Parwani et al. 2005). A gradual increase in the anesthetic dose produces a gradual worsening of short-term memory and a gradual shortening of the time-interval after which memories can be retrieved (Andrade et al. 1994). General anesthetics generally do not impair existing long-term memory (Bramham and Srebro 1989), since the inter-postsynaptic hemifusions responsible for them are well stabilized by different mechanisms. It was reported that sevoflurane (0.1 MAC), when administered for a specific time, enhances aversive memory formation in rats (Alkire et al. 2005). Since associative learning induces the enlargement of dendritic spines, allowing them to make close contact and hemifusions, sevoflurane at a very low dose can augment this mechanism, permitting the formation of the maximum possible number of specific inter-postsynaptic functional LINKs during a narrow window of time at a specific concentration.

## Sleep and unconsciousness

When the frequency of oscillating potentials is reduced both during sleep consciousness is altered (Alkire et al. 2008). Along with this, thalamo-cortical oscillating potentials differ during sleep from that in awake states (Steriade et al. 1993). The depth of unconsciousness (threshold for arousal) during sleep varies between different stages of sleep. Unconsciousness during sleep can be explained by the observed expansion of the ECM (Xie et al. 2013) that reduces the space occupied by the cellular components. This will exert lateral pressure on the dendritic spines that will increase the probability of them getting inter-LINKed, increasing the magnitude of the horizontal component contributing to the oscillating potentials. This will present as a slowing of oscillating potentials as seen in sleep.

## Cognitive defects and neurodegeneration

**Fig. 8:**
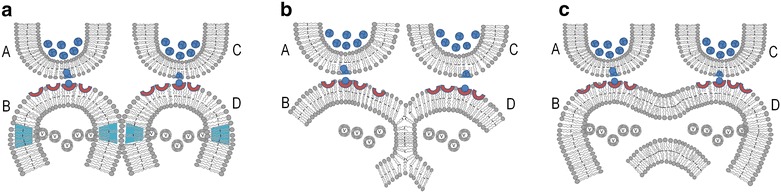
Reversible and irreversible changes induced by the insertion of anesthetic molecules to the lipid membrane. These changes are an extension of changes described in Fig. 5. **a** The insertion of an anesthetic molecule (an) within the lipid bilayer results in membrane expansion and formation of reversible partial hemi-fusion between postsynaptic membranes *B* and *D*. Once the anesthetic molecule is removed, this readily reverses back to normal state. **b** Complete hemifusion between postsynaptic membranes *B* and *D*. This is also a completely reversible process. However, prolonged maintenance of this state can lead to insertion of trans-membrane proteins across the hemifused membrane segment and stabilize this region for the duration of the life of that protein. Strong checkpoint mechanisms are expected to be present that prevent conversion of hemifusion to a fused state. **c** Inter-postsynaptic membrane fusion. Membrane hemifusion is an intermediate stage in the process of fusion. A well-conserved checkpoint mechanism that prevents conversion of hemifusion to fusion is expected to present at the postsynaptic membranes. Factors such as membrane composition changes, type and concentration of anesthetics or failure of checkpoint mechanisms can promote conversion of hemifusion state to fusion. Fusion occurring between the postsynaptic membranes is most likely an irreversible process and can trigger various neurodegenerative changes

An emerging consensus view on the possible link between Alzheimer’s disease and anesthesia is being examined following reports by several studies of a positive correlation between them (Baranov et al. 2009). Neurotoxicity following pediatric anesthesia has also received much attention (Ramsay and Rappaport 2011). These studies highlight the importance of verifying reversible partial membrane hemifusion as a mechanism of anesthetics. The anesthetic-induced changes expected to occur between the postsynaptic membranes are a spectrum of changes that range from close contact between the membranes by excluding the hydrophilic region to reversible partial and complete hemifusions. Hemifusion is an intermediate stage of the membrane fusion process. Strong checkpoint mechanisms are expected to be present at the level of the postsynaptic membranes that prevent conversion of hemifusion to a fused state. Since very high pressure is required to induce hemifusion, it can take place only in favourable conditions. However, various factors—such as membrane lipid compositional changes (Fuller et al. 2003), electrolyte changes, the presence of abnormal proteins, chemical molecules (Haque et al. 2001; Mondal and Sarkar 2011), the nature and concentration of anesthetics and the failure of checkpoint mechanisms that prevent conversion of hemifusion to fusion (Fig. 8)—can lead to deleterious consequences. Fusion between the postsynapses (dendritic spines) can lead to cytoplasmic content-mixing between two different neurons. The finding that gene expression profiles among the same neuronal types, such as CA1 pyramidal neurons, are different (Kamme et al. 2003) indicates that cytoplasmic content-mixing—even between similar cell types—can trigger cytotoxic consequences. These include dendritic spine loss and the triggering of cellular pathways that lead to apoptosis. All these changes produce changes similar to those that are seen in neurodegenerative diseases.

## Testing the present work

The following investigations can be undertaken to test whether the mechanism provided in the present work can explain how anesthetics work.

1. Disruption of the intactness of pachymeninges can prevent the external pressure from getting transduced through the CSF space to the paravascular space and finally to the ECM. This is expected to prevent the reversal effect contributed through the hydrostatic pressure.
2. The cellular changes of the anesthetic-induced inter-postsynaptic membrane functional LINK are a spectrum of changes, ranging from direct membrane contact to reversible, inter-postsynaptic membrane hemifusion restricted to very small areas of approximately 10 nm^2^. These inter-membrane interactions depend on the membrane lipid composition and the nature of anesthetic agents. Development of high-resolution imaging techniques that can resolve real-time changes at nanometer scales (Chen et al. 2014) will help with understanding reorganization, close contact, hemifusion and reversal of hemifusion at nanoscale domains of postsynaptic membranes.
3. Studies of the effect of anesthetic molecules on artificial membrane hemifusion process can be conducted. Role of membrane proteins and lipid composition on membrane interactions can also be studied.
4. The relationship between neurodegeneration and the use of anesthetics with different octanol/water coefficients, their MACs, duration of use and lipid membrane composition can be examined both by clinical studies and by laboratory experiments using peripheral blood cells or by using artificial membranes.

## Conclusion

The problem of understanding the mechanism of anesthesia has been persisted since the mechanism for consciousness remained unknown. Even though a framework for consciousness was put forward earlier (Vadakkan 2010), it was not until the discovery of the glymphatic system (Iliff et al. 2012) that a framework for a mechanism that can explain different findings in anesthesia research became possible. The mechanism presented here is not an alternative to the known mechanisms of action of different types of anesthetics that act on different membrane bound receptors; rather, it provides a common mechanism of anesthetics that can alter the net C-semblance proposed for consciousness. The mechanism that allow the anesthetics to get removed from the membranes through the paravenular system described in the present work is different from previously thought direct effect of pressure on enzymes (Moss et al. 1991). Changes at the substrate binding sites of enzymes along with partial molar volume changes described earlier (Imai et al. 2006) can occur along with the changes described in the present work. By exploring membrane interactions that can potentially lead to reversible membrane hemifusion changes, further verification of the anesthetic mechanisms can be explored. Evidence for the presented mechanism of anesthetics is expected to retrospectively contribute to our understanding of consciousness. The present framework should be treated as unproven until it is verified against further supporting evidence.

## Abbreviations

AMPA: α-amino-3-hydroxy-5-methyl-4-isoxazole-propionic acid
AMPAR: AMPA receptor
ARAS: ascending reticular activating system
CSF: cerebrospinal fluid
Dendritic spine: postsynapse or postsynaptic membrane
ECG: electroencephalogram
ECM: extracellular matrix
EPSP: excitatory postsynaptic terminal
GABA: gamma-aminobutyric acid
GluR: glutamate receptor
GluR1: GluA1 subunit of AMPAR
IILPS: islets of inter-LINKed postsynapses
LINK: inter-postsynaptic functional link
LTP: long-term potentiation
MAC: minimum alveolar concentration
NMDA: *N***-**methyl-d-aspartic acid
Postsynapse: postsynaptic terminal or dendritic spine
RF: reticular formation
SNAP: synaptosomal associated protein
Spine: dendritic spine (postsynapse or postsynaptic terminal)
SR: sensory receptor
